# Manufacturing and Mechanical Behaviour of Scalmalloy^®^ Lattice Structures: Experimental Validation and Model

**DOI:** 10.3390/ma18153479

**Published:** 2025-07-24

**Authors:** Ilaria Lagalante, Diego Manfredi, Sergio Balestrieri, Vito Mocella, Andrea El Hassanin, Giuseppe Coppola, Mariangela Lombardi, Paolo Fino

**Affiliations:** 1 Department of Applied Science and Technology (DISAT), Politecnico di Torino, Corso Duca degli Abruzzi 24, 10129 Torino, Italy; diego.manfredi@polito.it (D.M.); paolo.fino@polito.it (P.F.); 2 Interdepartmental Center of Integrated Additive Manufacturing (IAM@PoliTo), Politecnico di Torino, Corso Castelfidardo 51, 10129 Torino, Italy; 3 National Research Council, Institute of Applied Science and Intelligent System, Via Pietro Castellino 111, 80131 Napoli, Italy; sergio.balestrieri@na.isasi.cnr.it (S.B.); vito.mocella@na.isasi.cnr.it (V.M.); giuseppe.coppola@cnr.it (G.C.); 4 Department of Chemical, Materials and Industrial Production Engineering, University of Naples “Federico II”, Piazzale Vincenzo Tecchio 80, 80125 Naples, Italy; andrea.elhassanin@unina.it

**Keywords:** laser powder bed fusion, lattice structures, aluminium alloy, Scalmalloy^®^, lattice design, process optimisation, compression tests

## Abstract

This study investigates the influence of process parameters on the fabrication and mechanical performance of Scalmalloy^®^ lattice structures produced via laser powder bed fusion (PBF-LB) and their mechanical responses at different cell size. A full-factorial design of experiments was employed to evaluate the effect of scan speed, hatch distance, and downskin power on internal porosity and dimensional accuracy. Regression models revealed significant relationships, with optimised parameters identified at a scan speed of 700 mm/s, hatch distance of 0.13 mm, and downskin power of 80 W. Mechanical characterisation through tensile tests of bulk samples and compression tests of lattice structures highlighted the strengthening effects of the heat treatment. Experimental data on quasi-elastic gradient and yield strength were compared to predictions from the Ashby–Gibson model, revealing a partial agreement but noticeable deviations attributed to cell geometry and manufacturing defects. The scaling laws observed differed from the classical model, particularly in the yield strength exponent, indicating the need for empirical models tailored to metallic lattices. This work provides key insights into the optimisation of PBF-LB parameters for Scalmalloy^®^ and underlines the complex interplay between process parameters, structural design, and mechanical behaviour.

## 1. Introduction

Additive manufacturing (AM) has broadened the industries to new, previously unthinkable possibilities. Mechanical properties are improved thanks to a peculiar microstructure characterised by very fine grains and supersaturated solid solutions, which is further improvable by defined heat treatments. But most of all, the layer-by-layer technology overcomes all the design restrictions of traditional processes, allowing the building of complex and innovative designs with very few limitations, making it possible to build directly complex internal channels and advanced geometries.

Among the additive technologies, laser powder bed fusion (PBF-LB) has shown great potential for the production of metal parts thanks to improved mechanical properties and good surface roughness compared to other powder bed technologies; yet it is still unsatisfactory compared to more traditional manufacturing routes such as machining [1,2]. It is a so-called “cold process”, meaning that the powder bed is not preheated like in electron powder bed fusion, and thus, the difference in temperature before and after melting is very significant. If the high temperature difference from one side generates a very high cooling rate, around 105–106 K/s, with a consequent very fine microstructure and improved but anisotropic mechanical performance, from the other side, it causes high thermal gradient and thus significant stresses, necessitating supports in order to better accommodate heat diffusion and decreasing the stresses [3].

One of the most promising and employed design achievements obtainable through AM is the building of lattice structures. These features, also called cellular or trabecular structures, are similar to open-cell foams, although they are regular and highly controlled, allowing for customised mechanical properties and structural behaviour [4,5]. There can be different types of lattice structures; one of the most common types are the strut-based ones, where the nodes of the repeated cells are connected by beams called struts [4]. Other than that, there can also be triply periodic minimal surfaces (TPMS), which are created from mathematical equations [6], and bio-mimetic ones, which are geometrically challenging designs inspired by natural elements such as sea sponges [7] or beetle shells [8]. Lattice structures have been widely introduced for a variety of different applications, including the lightweighting of parts by substituting lattice to bulk [9], typical automotive and aerospace applications like heat exchangers or heat pipes [10,11], and biomedical devices thanks also to improved osteointegration [12,13], among others.

The final mechanical properties of lattice structures depend on different aspects, as explained by Ashby and Gibson. In his “The properties of foams and lattices” [5], containing discussions that are further expanded in [14,15], Ashby explained that the lattice mechanical behaviour is not only influenced by the material from which the lattice is made and responsible for the intrinsic properties of the solid material, but it is also influenced by the design used for its cells. Although initially developed for foams, the laws discussed by Ashby and Gibson have been extended to trabecular structures and have been frequently applied to describe the compressive behaviour of lattice structures produced by AM technologies [4,16,17]. In particular, great influence has been attributed to the cell topology and shape and to the relative density, which is the ratio of the density of the cellular structure to the density of the solid material [16,18]. The cell design greatly influences the dominating behaviour during the compressive tests. Struts can indeed be seen as Euler–Bernoulli beams, and they can tend to bend or to stretch depending on the constraints in the cell [4]. Furthermore, the mechanical properties, in particular the compressive stress and the static modulus, have been reported to show a positive power relationship with the relative density, allowing mechanical behaviour predictions through the Ashby–Gibson model, where the nature of this relationship depends on the dominant behaviour [5,19].

Al alloys have been often employed also in lattice structure applications [20,21,22] thanks to their low density, excellent thermal conductivity, and good corrosion resistance. One of the most investigated Al alloys processed by PBF-LB is surely AlSi10Mg [23,24,25], which is a highly feasible AM Al-Si alloy thanks to its narrow solidification interval, having a near eutectic composition [25,26,27]. This alloy is also extensively employed for lattice production [20,21,22]. For instance, Ferro et al. [28] studied the use of trabecular structures for an anti-icing sandwich panel built by PBF-LB using AlSi10Mg, with improved stiffness and light weight. Another interesting Al alloy for PBF-LB is Scalmalloy^®^. Introduced by Airbus APWorks^®^ for aerospace applications, Scalmalloy^®^ is characterised by an impressive strength-to-weight ratio that has contributed to its widespread adoption. For instance, it has also recently been introduced as one of the allowed materials for Formula 1 applications, opening a new route in the racing industry [29]. Scalmalloy^®^ exhibits outstanding mechanical properties due to its peculiar microstructure and the addition of scandium, as extensively reported in the literature [30,31,32,33,34]. In the as-built condition, it typically presents a bimodal grain structure, with extremely fine equiaxed grains along the melt pool boundaries and larger, columnar grains within the melt pool. This configuration enhances grain boundary strengthening in accordance with the Hall–Petch relationship. Grain refinement is further promoted by the presence of finely dispersed, fully coherent nanoscale Al_3_Sc precipitates, which act as nucleation sites due to their excellent lattice match with the aluminium matrix. These precipitates not only facilitate grain refinement during solidification but also effectively inhibit grain coarsening by pinning grain boundaries during subsequent heat treatment. Heat treatments in the range of 325–350 °C for 4–10 h have been reported to promote the controlled precipitation of these nanoscale Al_3_Sc phases without reporting any grain coarsening [29], although a heat treatment at 325 °C for 4 h is commonly recommended by powder suppliers [35]. The controlled precipitation of Al_3_Sc during heat treatment significantly contributes to precipitation hardening by impeding dislocation movement, an effect that is further improved by the coherency between precipitates and matrix; at the same time, these phases preserve the fine-grained microstructure established during the PBF-LB process by inhibiting their coarsening. This dual strengthening mechanism results in a notable increase in yield and tensile strength after heat treatment, albeit at the expense of reduced elongation at fracture [30,32]. Notwithstanding the massive interest from industrial and academic research, the use of Scalmalloy^®^ for lattice structures has been reported in a very limited amount of works to the author knowledge [36,37,38].

Even if lattice structures have great potential for many applications, the final quality of the structures might still be an issue. Indeed, their geometry is usually complex, with a high number of overhanging features, which have been reported to be particularly problematic for the final quality of both internal densification and surface quality, in particular at the downskin [39,40]. The downskin refers to the initial layers of downward-facing surfaces, which are built by melting powder directly onto the loose powder bed without prior solid support. These layers tend to have a difficult heat dissipation due to the lower thermal conductivity of the powder that surrounds them, leading to worse surfaces and major defects. Lin et al. [41] thoroughly investigated how the lower thermal conductivity of powder and subsequent worst heat dissipation translates to a high heat accumulation, which causes excessive melt pool melting and a massive attachment of powder particles to the struts, leading to huge dross formations and resulting in poor surface quality and dimensional accuracy. The choice of process parameters must, therefore, be carefully and efficiently made in order to avoid or at least minimise these undesired effects. For instance, De Pasquale et al. [42] analysed the parameter effects on vertical and horizontal struts, demonstrating that a too-high volume energy density (VED) causes rough and irregular profiles, whereas a too-low one leads to weak and damaged struts. Furthermore, both Qiu [43] and Han [44] reported how higher scan speeds can improve the surface quality and dimensional accuracy of thin AlSi10Mg struts.

Nonetheless, the effects of the process parameters on the resulting lattice are usually overlooked or simplified due to the complexity of the factors, without focusing on how the individual parameters affect the process differently or influence each other. However, statistical tools can help to observe and understand these effects better.

The aim of the present work is to analyse these effects for the production of lattice structures produced by PBF-LB using Scalmalloy^®^. A detailed statistical analysis of the process parameters is provided, which enables a more precise and accurate observation of the cause (process parameters)–effect (part quality) relationships, contributing to a better understanding of the process optimisation. This includes investigating aspects such as downskin power, which is often overlooked but has a significant impact on lattice quality. The analysis focused on three different parameters and how each of them, alone or in combination, influenced the final density and dimensional accuracy in different ways. A predictive model was then found and used to define the optimised parameter set that gave the best compromise between density and dimensional accuracy. With this knowledge, samples were built to study the lattice compressive behaviour and the influence of the heat treatment on the final behaviour of Scalmalloy^®^ lattices. Indeed, as previously stated, even though Al alloys have been extensively explored for lattice applications, the use of Scalmalloy^®^ for lattice structures has been rarely explored. The present work adds new insights by providing the compression mechanical properties of Scalmalloy^®^ lattice structures either before or after heat treatment, highlighting the potential of this alloy for lightweight applications while offering process–structure–property mapping that can support further design and optimisation efforts in the AM of advanced lattice materials. Furthermore, two more sample types with the same cell design but different strut sizes were tested to verify the relationship between resulting mechanical properties and relative density, as suggested by the Ashby–Gibson model.

## 2. Materials and Methods

### 2.1. Samples Production

The specimens were manufactured using a Scalmalloy^®^ powder provided by LPW Technology Ltd. (now Carpenter Additive, Philadelphia, PA, USA), a commercial supplier. The powder exhibits a density of 2.67 g/cm^3^ and has a chemical composition provided by the powder producer; it is displayed in Table 1.

**Figure 1:**
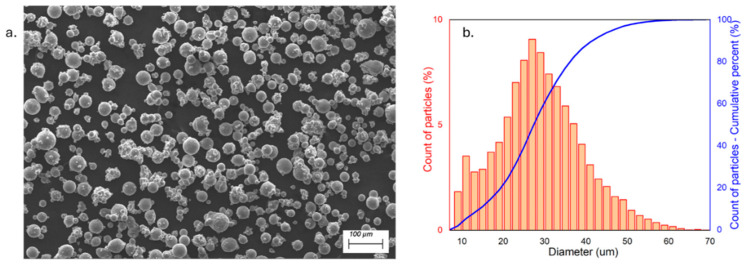
(**a**) SEM of Scalmalloy^®^ powder; (**b**) PSD of particles.

The particle size distribution (PSD) was defined using a Morphologi 4 (Malvern Panalytical, Malvern, UK), assessing D10, D50, and D90 percentiles to 14.15, 27.3, and 41.5 µm, respectively (Figure 1b).

The instrument was also used to define the powder circularity: average circularity, measured on almost 10,000 particles, was assessed at 0.92, implying a very good circularity, though not excellent. Further examination through scanning electron microscopy (SEM), shown in Figure 1a, highlights that the particles possessed an optimal sphericity, but a significant quantity of satellites was also evident. Few irregular and big agglomerates could also be spotted, decreasing the overall circularity.

The powder was then processed by an EOSINT M270 Dual-mode system (EOS GmbH, Munich, Germany) in an argon atmosphere, keeping the oxygen level below 0.1%. The system employs a Yb-fibre laser with a wavelength of 1063 nm (red laser). The laser works in continuous mode and has a maximum power of 200 W and a spot size of 100 µm.

**Figure 2:**
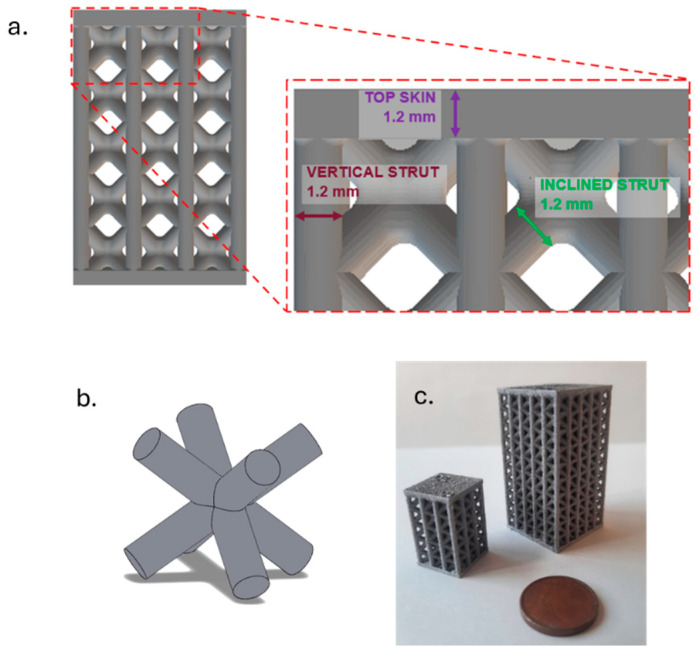
Lattice structures used in the study: (**a**) highlight of main features; (**b**) focus of the BCC cell; (**c**) built sample, in particular the one used for the DOE analysis (smaller one, **left**) and the one used for compressive tests (bigger one, **right**).

The design of the lattice produced was defined on the basis of the indication of Virgillito et al. [45], who designed a lattice structure in AlSi10Mg. The cell used was a body-centred cubic one (BCC, Figure 2b) with vertical struts in the perimetric outer cells. The struts have a diameter of 1.2 mm, and each cell has a size of 4.0 mm.

In addition, a skin sized 1.2 mm was built at the bottom and top of the lattice as a support to the structure (Figure 2a).

The relative density, which can also be expressed as solid material volume fraction, i.e., the ratio between the volume of the lattice and the one of the solid that encloses it, was 43%. Two different geometries were produced. The first one had three cells along the X-Y direction and five along the Z direction and was used for the process optimisation. The second one had 5 × 5 × 10 cells and was used for compressive tests (Figure 2c).

### 2.2. Design of Experiments and Characterisation Tests for Surface Texture and Dimensional Accuracy

**Figure 3:**
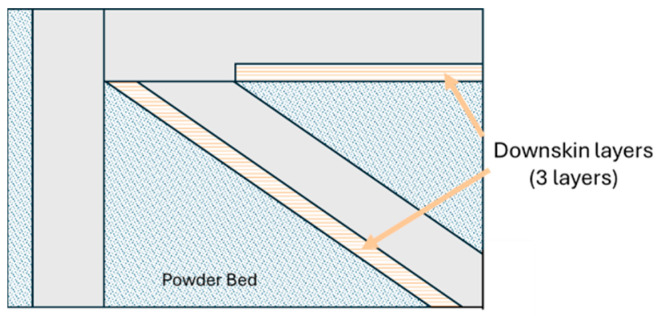
Representation of downskin layers.

In order to identify the best parameter set that optimised both internal density and dimensional accuracy, a full-factorial design of experiments (DOE) was developed and analysed. However, some process parameters were kept as constants for all experimental sets, using the conditions established for the production of bulk Scalmalloy^®^ samples on the same machine. In particular, the core infill laser power was set to 195 W, the layer thickness to 0.03 mm, and the scan strategy to a 67° rotation for all samples. The platform was heated to 100 °C, and samples were produced directly on the platform with a surplus of material of 0.5 mm. Samples were later cut from the substrate via electrical discharge machining, removing the material surplus and ensuring coplanarity between the skins. Particular attention was given to the power applied to the downskin layers, which are the layers directly melted on powder (Figure 3). Apart from laser power, the other parameters were not changed depending on the layers orientation.

The full-factorial DOE analysed three different factors, i.e., scan speed and hatch distance used in the whole part and the power used in the downskin layers, referred to as downskin power. Three levels were used for downskin power and hatch distance and four levels for the scan speed, as depicted in Table 2, for a total of 36 sets of parameters. Their values were picked starting from the parameter set optimised for bulk Scalmalloy^®^ produced on the same machine, i.e., 195 W, 0.17 mm, and 400 mm/s. The level values were chosen to explore a technologically relevant window where measurable effects could be observed while avoiding conditions known to induce large process instability. The parameter range was deliberately constrained within these practical limits to investigate process parameter influences and interactions under conditions relevant to industrial PBF-LB of Scalmalloy^®^. This ensured that the significance analysis remained applicable to real processing windows, while the use of statistical methods enabled the detection of subtle parameter effects, enhancing the robustness of the analysis. Prior to any analyses, all samples were ultrasonically cleaned in ethanol for five minutes to remove any loose powder left after the job production. For all samples, dimensional accuracy and internal density were evaluated.

**Figure 4:**
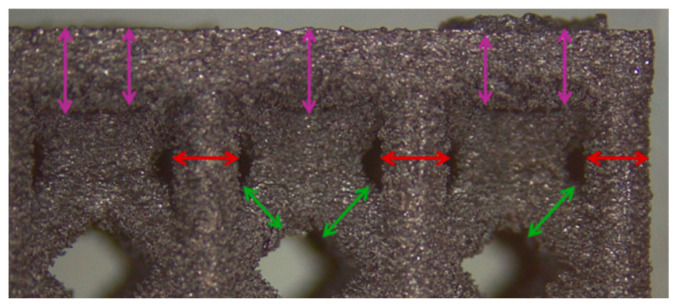
Features measured for the DOE analysis, i.e., inclined struts (green arrows), vertical struts (red arrows), and top skin (purple arrows).

Dimensional accuracy was evaluated through image analysis. Several images of the external cells were taken using a stereomicroscope (Leica EZ4 W, Wetzlar, Germany) at a magnification of 16× and used to measure the strut diameter. The strut dimensions were quantified using ImageJ2 software (National Institutes of Health, Bethesda, MD, USA), which allowed the measurement of the external features. In particular, three features, i.e., the peripherical vertical struts, the inclined struts, and the top skin, were evaluated. At least ten random struts were picked for each feature, and a total of 30 measurements per feature were collected by measuring the strut thickness (Figure 4). After collecting all data, their average was measured and compared to the theoretical 1.2 mm strut dimension, resulting in a dimensional deviation from the ideal feature. This deviation was then used in the DOE analysis.

Additionally, surface roughness parameters were also measured at multiple locations on the selected samples using a confocal microscope (Leica DCM 3D, Wetzlar, Germany), which allows for non-contact, high-resolution surface profiling with vertical resolution in the nanometre range. This technique is particularly suitable for analysing complex geometries without requiring sample preparation. To assess surface variability across different structural elements, some distinct regions were analysed for each sample: a 1 mm × 0.19 mm area on the horizontal rod (downskin area of the top skin) and a 0.2 mm × 0.3 mm area on the downskin of the diagonal struts. From each of these regions, key roughness parameters were extracted, including root mean square height (Sq), which gives a statistical value of the peak variability; maximum height (Sz), which is the distance between the largest peak and the largest valley; arithmetic mean height (Sa), a generally used parameter for the average surface roughness; maximum valley depth (Sv); and maximum peak height (Sp), all defined by the ISO 25178 standard [46,47]. This systematic approach ensured a comprehensive characterisation of the surface topography across the entire lattice structure.

Sample density was then evaluated using the Archimedes method by measuring the sample weight in air and its weight in a reference fluid, allowing the calculation of density based on the displaced volume determined via the Archimedes principle. Therefore, using the Archimedes balance at a temperature of 20 °C, the sample mass was measured in both air and ethanol; ethanol was chosen as reference fluid instead of distilled water because of a better wettability, which allowed more accurate results [48]. Samples were left in ethanol for 15 min in order to ensure more accurate results and stable wetting. Three measurements were taken for each sample. Following the Archimedes principle, the material density of each lattice was therefore measured as

(1) $$\rho =\frac{m_{A}}{m_{A}-m_{E}}\left(\rho_{E}-\rho_{A}\right)+\rho_{A}$$

where *m_A_* and *m_E_* are the samples mass weighted in air and ethanol, respectively, and *ρ_E_* and *ρ_A_* are the density of ethanol (0.79 g/cm^3^) and air (0.0012 g/cm^3^), respectively [48]. It is noted that the air density, while reported for completeness, is significantly lower than the density of Scalmalloy^®^ and does not substantially affect the accuracy of the measurement. The apparent density was instead measured as a ratio between the measured material density of the lattice and the material theoretical one, i.e., 2.67 g/cm^3^. At last, porosity can be measured as the complementary percentage of the apparent density. To validate the porosity results, selected samples were mounted within the cold resin and cut along the XZ plane and then polished and observed using an optical microscope (Leica DMI 5000 M, Wetzlar, Germany). The resin allowed the sample to be cut without damaging the thin struts.

**Figure 5:**
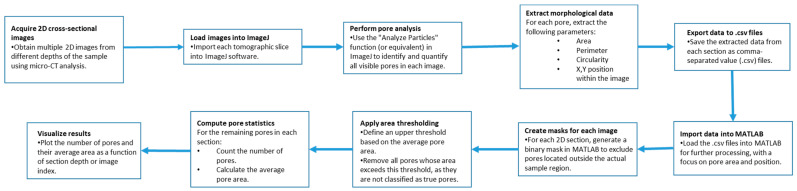
Algorithmic workflow for the analysis of micro-CT cross-sectional images: pore features are extracted in ImageJ, then processed in MATLAB through masking and area-based thresholding, leading to the quantification of pore count and average area per section.

Furthermore, on the optimised sample, micro-computed tomography (micro-CT) was performed in order to observe porosity distribution. In typical micro-CT systems, an X-ray source emits a cone beam that passes through the sample, with attenuation depending on the material’s density and composition. The acquired projection data are then reconstructed using filtered back-projection or iterative algorithms to generate detailed 3D representations. The micro-CT analysis reported in this manuscript was performed using a Bruker Skyscan 1273 (Billerica, MA, USA), employing two scanning protocols to optimise spatial resolution and contrast. Samples were scanned using an X-ray beam generated at 110 kV and 232 μA, filtered with a 0.5 mm Al filter, and with an exposure time of 270 ms. The transmitted radiation was captured by a digital detector (Varex 2315-S/N 55251, Varex Imagening, Walluf, Germany) with a pixel size of 80 μm, positioned at a relative distance of 121 mm from the X-ray source. This setup produced a series of two-dimensional radiographic projections. During scanning, the sample was rotated on a motorized stage through 360°, in steps of 0.15°, allowing projection data to be acquired at multiple angles. A total of 1666 2D scans were acquired at vertical intervals of 8 μm, covering the entire sample height. These projections were then computationally reconstructed into virtual cross-sections at various depths, ultimately yielding a high-resolution 3D model of the sample with a final spatial resolution of 8 μm. The porosity analysis (Figure 5) was performed by initially processing X-ray images using ImageJ software.

**Figure 6:**
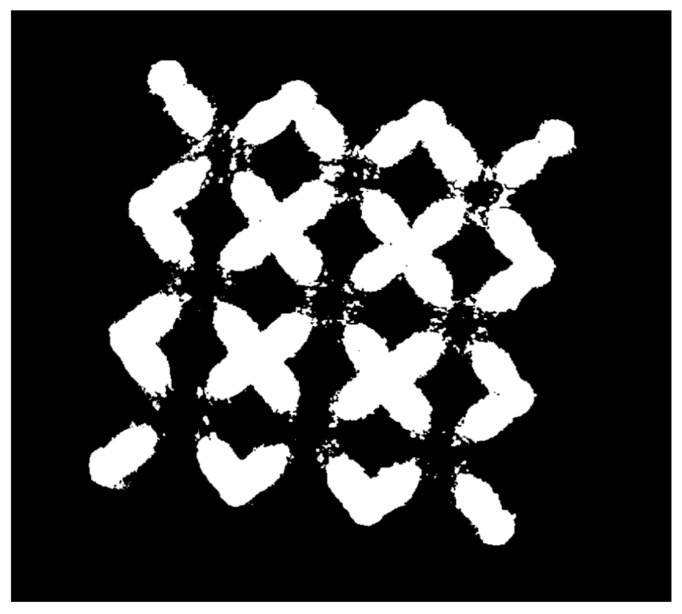
Binary Mask of a sample processed by the algorithm.

Specifically, an automated algorithm was employed to analyse all pores identified in each micro-CT cross-section, extracting their main geometric features, including area, perimeter, major axis length, minor axis length, and circularity (defined as the ratio of minor to major axis length, which quantifies how closely the pore cross-section approximates a perfect circle). All extracted data were exported as .csv files and subsequently processed using a custom MATLAB script. Using this script, pores were filtered by area using a size threshold to eliminate potential segmentation artifacts from the ImageJ analysis. Additionally, a spatial mask (Figure 6) was applied to remove any erroneously identified pores located outside the physical boundaries of the sample.

The resulting dimensional accuracy and density were evaluated by multiple regression using the statistical commercial software Minitab [49]. As previously introduced, the DOE used a factorial design with multiple levels, with three factors (downskin power, hatch distance, and scan speed) and a total of four responses (porosity and the dimensional deviation from the CAD of top skin, inclined struts, and vertical struts size, referred to as simply top skin, inclined strut, and vertical strut, respectively), as summarised in Table 2. The multiple regression analysis was used to evaluate the influence of the parameters as well as their interactions on each response.

An important element of the model is the *p*-value, which describes the significance of the influence of each factor on each response. *p*-values lower than the fixed target one indicates that the factor has a significant influence on the response: in this work, the target *p*-value was fixed to 0.05, which indicates a 95% confidence level. Each model has a coefficient of determination (R^2^) that describes the model ability to fit experimental data; the closer to 100%, the more reliable the model is, though having nonlinear terms in the equation tends to decrease it. Then, the multiple regression model is described by a function that correlates each factor that has a significant influence on the response to the response itself [50]. The general function of a response y, considering two factors x_1_ and x_2_, is defined as

(2) $$y=b_{0}+b_{1}x_{1}+b_{2}x_{2}+b_{3}x_{1}^{2}+b_{4}x_{2}^{2}+b_{5}x_{1}x_{2}$$

where *b*_0_, i.e., the intercept coefficient or constant term, and *b_n_* are the regression coefficients multiplying the variables, indicating the change in the response for a one-unit change in the corresponding factor, and the direction of this relationship. The model can also describe nonlinear relationships (*x_n_*^2^) and interactions between different factors (*x_n_x_m_*). Factors that did not show any significant relationship were removed from the model, even if their interaction showed significance. In order to improve the quality of the 3D surface plot, MATLAB was used to plot the equation model defined by Minitab. Finally, the model identifies an optimised parameter set that ensures the best-compromised optimisation of all responses by fixing a desired target for each response [50,51,52].

### 2.3. Mechanical Tests

Following ISO 13314-2011 [53], compressive specimens with struts of 1.2 mm were tested at compression at room temperature, with a testing speed of 1 mm/min. The samples employed for the tests consisted of lattices with 5 × 5 × 10 cells, resulting in an overall size of 21 × 21 × 41 mm^3^. They were tested in both the as-built condition (AB) and the heat-treated one (HT), employing a heat treatment at 325 °C for 4 h as typical for Scalmalloy^®^ [30]. At least two samples per type were tested.

Then, samples with the same cell design but two different strut sizes were tested until reaching a deformation of 15% if failure had not taken place yet. The test results were analysed for verifying the relationship between the relative densities and the compressive stress and elastic modulus, according to the Ashby–Gibson model. In particular, compressive stress and elastic modulus were defined as compressive offset stress or lattice yield stress (σ_y_) and quasi-elastic gradient ($\tilde{E}$), respectively, measured according to ISO 13314-2011. The three strut sizes of different sample types were 0.8, 1.0, and the reference 1.2 mm, changing the relative density to 24, 33, and 43%, respectively.

To measure the material yield strength and elastic modulus required for verifying the Ashby–Gibson model, tensile tests were conducted in both conditions, AB and HT, in accordance with ASTM E8. Flat specimens were built horizontally, meaning the longitudinal axis was parallel to the building platform. The tests were carried out at room temperature, with a preload of 3 MPa and a test speed of 0.008 1/s, and were repeated three times.

## 3. Results and Discussion

### 3.1. Process Parameters Analysis and Optimisation

#### 3.1.1. Influence of the Parameters on the Porosity

**Figure 7:**
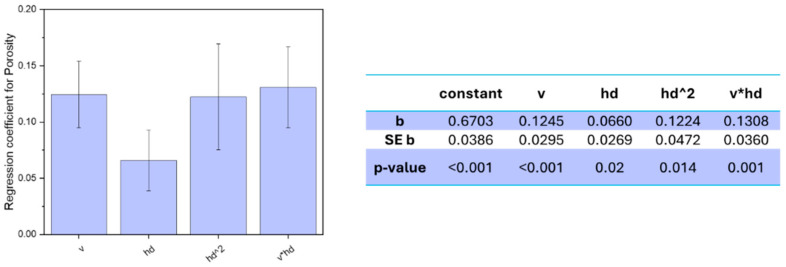
Regression coefficients for the porosity response model, with standard error ER and *p*-value.

The regression coefficients of the model for porosity response are depicted in Figure 7.

All the factors included in the model showed a significant influence on the porosity level, i.e., a *p*-value lower than 0.05, and the final model had an R^2^ of 59%, which implies that the factors influence is properly described by the model. However, it must be highlighted that the measured porosities were between 0.44 and 1.28% for all sets of parameters, which means that, either way, all sets of parameters showed a decent density without any major deficiency.

Analysing the regression coefficients of the model equation (Figure 7), it was observed that the downskin power did not show any significant relationship to the final density of the part, being the only factor not included in the model. On the other hand, both hatch distance and scan speed, other than their interaction, showed a linear dependency on the final porosity. In addition, the hatch distance showed a quadratic relationship to the porosity response. Out of the four coefficients having significant relationships, three had very similar high regression coefficients, meaning that all three had a great influence on the final part, whereas the fourth, the linear hatch distance, had a slightly lower impact on the response. Scan speed showed a high correlation to the resulting porosity, with a coefficient of 0.1245 and a *p*-value less than 0.001. The dependency is linear, and the positive sign of the coefficient suggests that increasing the scan speed led to an increase in porosity. Indeed, the scan speed optimised for the bulk alloy was 400 mm/s, and increasing the speed decreased the energy density during the melting process, negatively affecting the densification.

**Figure 8:**
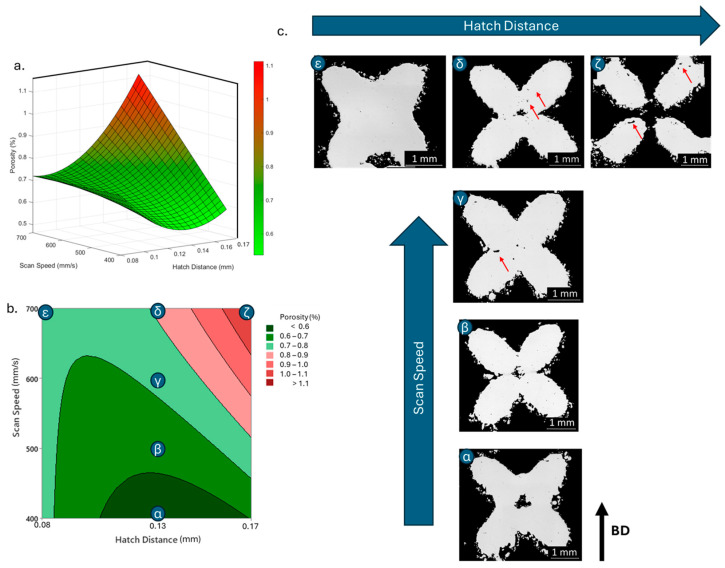
Porosity response to scan speed and hatch distance in 3D (**a**) and contour (**b**) plot; (**c**) images of density of defined samples with porosities highlighted by red arrows.

The lower energy decreases the temperature inside the melt pool, generating a thinner and shallower melt pool, and might not be enough to fully melt the scanned material. This can lead to irregularities in the densification, such as unmelted powders inside the part, known as lack-of-fusion [23,54]. This is also consistent with what was already found by Qiu et al. [43] for AlSi10Mg thin struts. They also reported a decrease in density correlated to an increase in scan speed, up to more 2% of porosity when increasing from 1000 to 4000 mm/s. Figure 8c shows that by increasing the scan speed while maintaining the other parameters unaltered, a higher number of internal porosities can be spotted, though the overall densification still appears good, with a density over 98.5%.

Besides scan speed, hatch distance showed also a significant influence on the final porosity. Both a linear and a quadratic relationship were displayed, with a confidence level over 98% and a positive correlation in both cases, though the quadratic relationship showed a double coefficient compared to the linear one, implying a more important effect on the response, i.e., 0.1224 and 0.066, respectively. As aforementioned for the scan speed, the overall energy density decreased when the hatch distance increased, thus reducing the solidification rate and increasing the occurrence of imperfections in the densification process. Indeed, the linear influence of the hatch distance on the final density of aluminium alloys was already reported, with higher porosities for higher hatch distance [23,54]. On the other hand, a quadratic relationship has seldom been observed. Riener et al. [51], who observed it for an Al6182, ascribed the observed quadratic trend over the more common linear one to a narrower range of hatch distances tested in the DOE. However, it can be otherwise explained. A further analysis of the relationship highlights that the interaction between hatch distance and scan speed also has a significant influence on the porosity response, with a *p*-value of 0.001 and the highest regression coefficient of 0.1308. Observing the surface plot in Figure 8, the quadratic relationship of the hatch distance becomes very evident, especially at lower scan speeds. Indeed, porosity increases with hatch distance at higher scan speed, reaching its highest measured value at the lowest energy density, when both hatch distance and scan speed are at their highest (Figure 8c). Nonetheless, a different trend is observed at lower scan speeds. Though still increasing porosity at higher hatch distance, the lowest hatch distance also showed a negative effect on the densification, with the minimum porosity reached for middle hatch distance values.

It can be presumed that the low hatch distance generates too-high energy when combined with the low speed; this high energy might cause overheated spots and the onset of unwanted phenomena such as keyholes, decreasing the final densification [55]. This observation might explain the predominance of a quadratic behaviour over a linear one.

#### 3.1.2. Influence of the Parameters on the Dimensional Accuracy

Dimensional accuracy was evaluated by measuring the deviation of the struts from the ideal size, i.e., 1.2 mm. In general, poor surface quality was observed in all samples, with a high number of attached particles on the surface, which is typical of untreated PBF-produced surfaces [56], especially for aluminium alloys that usually show worse surface quality compared to other alloys [57,58]. In addition, drosses were evidenced in many samples, though at different entities depending on process parameters. Dross formation is indeed an undesired but inevitable phenomenon that occurs on overhanging structures, whose nature and physical causes have been investigated but are yet to be fully understood [40,41]. Three different features were analysed, and their deviation from the CAD dimensions was measured and used in the model: the diagonal strut, the vertical strut, and the top skin. The measurement results reveal that the greatest deviation from the ideal 1.2 mm size occurred in the top skin, with a minimum deviation of 0.142 mm and a maximum of 0.469 mm, whereas the inclined struts exhibited deviations ranging from 0.083 mm to 0.382 mm. Regarding the vertical struts, this feature showed the most dimensionally accurate results, with a maximum deviation of 0.158 mm and in some cases even a slightly thinner strut, deviating −0.006 mm from the ideal 1.2 mm. It is also noteworthy that the standard deviation is generally higher for the top skin measurements compared to the other two features, suggesting greater irregularities. Furthermore, the vertical struts exhibit an overall lower mean deviation but a relatively large standard deviation relative to the mean value, reflecting a wide scatter in the measurements. This variability ranges from very accurate dimensions, showing almost zero deviation from the nominal size, to larger deviations likely caused by process-induced variations, such as local defects, surface roughness, or measurement uncertainties.

These observations are aligned with the physical mechanism happening during the PBF-LB process. Indeed, the heat introduced in the laser track during the melting process tends to dissipate effectively by conduction over the previously melted layers, which are already in a solid state. However, overhanging features, especially for very low building angles, have limited connected area to the solid material that can suffice as a heat conductor. Thus, most heat is dissipated through the nearby low-conductive powder, which causes a slowdown of the cooling rate, and powder is also sintered or fully melted in the process, causing the appearance of major defects on part of the surface, such as drosses. This is particularly critical for horizontal features such as the top skin, which are completely in contact with the powder bed, causing the downskin to have a very high surface roughness and more defects [59,60,61]. Focusing on thin features such as lattice struts, a significant increase in their dimensions has been reported when decreasing the building angle because of the aforementioned phenomena. The effect is even more pronounced for bigger designed strut thicknesses because of the bigger area to get melted; thus, higher heat that has to be dissipated over powder [56,60].

**Figure 9:**
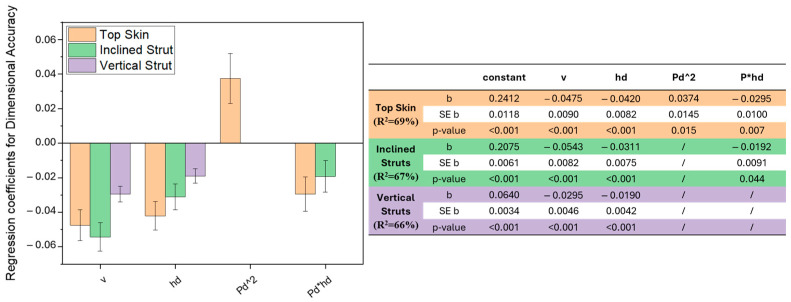
Regression coefficients for the dimensional accuracy response model, with standard error ER, *p*-value and also model coefficient of determination: top skin (orange), inclined struts (green), vertical struts (purple).

A model was defined for all three features, with regression coefficients and coefficient of determination reported in Figure 9. All models exceeded 65% of R^2^, suggesting a decent fitting of the model to the measurements. It can be noted that the top skin shows not only the highest R^2^, meaning the best fitting, but also the highest regression coefficients, indicating a higher influence of the parameters on the feature.

All three features show a higher relationship with the scan speed; in all cases, a linear increase in the scan speed values is correlated to a decrease in the dimensional deviation and thus an improvement in the dimensional accuracy. The same effect could be revealed when increasing the hatch distance, which indeed has a negative regression coefficient in all three models. It can also be observed that the hatch distance has an effect on the top skin and inclined struts also in relation to the power used in the downskin layers. As could have been expected, downskin power does not affect the vertical struts since it is not present in the feature, which is always built on already solidified metal with no overhanging surfaces. Finally, the top skin showed a correlation to the downskin power in a quadratic relationship.

As previously noted, increasing scan speed and hatch distance leads to a linear decrease in dimensional deviation (Figure 9). It is important to highlight that volumetric energy decreases as scan speed or hatch distance increases. This observation demonstrates that the final dimensional accuracy is positively influenced by the lower energy applied during the process, as reduced energy minimises excessive thermal accumulation and consequent defects.

On the contrary, a high presence of major drosses was observed for samples produced at low speed and low hatch distance, which confirms the detrimental impact of excessive energy. This phenomenon can be attributed to the higher temperatures risen in the melt pool due to the higher energy employed, which increases the infiltration of the melt pool in the surrounding powder bed [40].

The spreading of the melt pool, exacerbated also by the increased flowability caused by the higher temperatures, induces the formation of drosses and irregularities in the downskin of overhanging features and attached powder on all the surfaces. This effect is also very evident at the bottom of the nodes, where the inclined struts merge: indeed, the thermal effects of the four struts affect the whole area, generating big drosses and major powder attachment, aggravated by the gravity effect. The same defect at nodes was already reported by Salem et al. [62], who referred to it as sagging, a name taken from the welding nomenclature. Lowering the energy by increasing scan speed and hatch distance allows to mitigate the formation of these defects thanks to a better heat distribution that avoids any overheating. However, surface defects related to low energy have also been reported in the literature; for instance, Maamoun et al. [54] reported a worse surface quality for higher hatch distance due to a low overlapping of consecutive scan tracks. However, no such effect was observed with the experimental data used in the present study, presumably due to a narrower parameter range used that did not cause an energy low enough to cause such defects.

**Figure 10:**
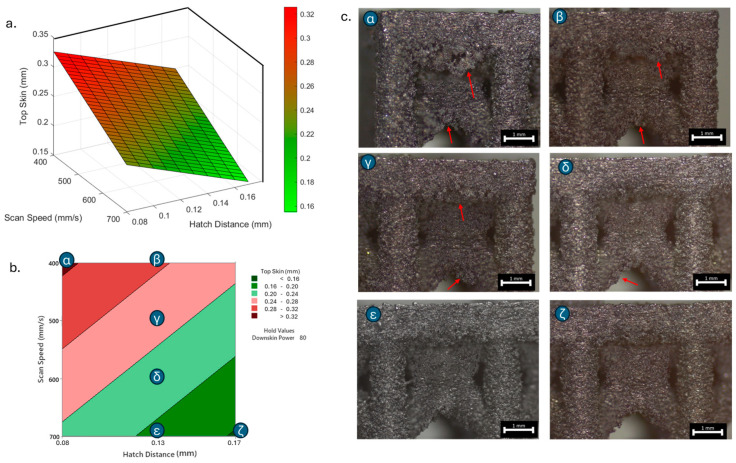
Top skin response to scan speed and hatch distance at constant downskin power of 80 W in 3D (**a**) and contour (**b**) plot; (**c**) images of defined samples with surface defects highlighted by red arrows.

As previously stated, the surface defects were especially noticeable at the top skin due to the predominant interaction with powder that worsened the thermal accumulation and caused the formation of massive drosses, especially at low hatch distance and scan speed (Figure 10).

**Figure 11:**
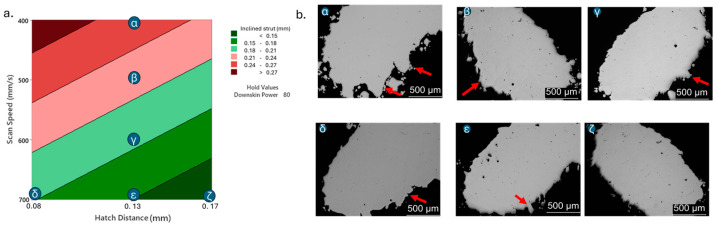
Inclined strut response to scan speed and hatch distance at constant downskin power of 80 W in contour plot (**a**); (**b**) images of defined samples with surface defects highlighted by red arrows.

Figure 11 also shows the effect of the different parameters on the sectioned inclined struts: infiltration can be observed when low hatch distance or scan speed are used, resulting in an uneven and irregular downskin surface.

On the other hand, the higher the scan speed and hatch distance used, the better the resulting surface, with just minor irregularities. The same trend was detected for the vertical struts, with a decreasing deviation from the ideal size when increasing hatch distance and scan speed. However, it must be noted that a negative deviation was measured at the highest scan speed, 700 mm/s, and hatch distance, 0.17 mm, meaning that the strut was slightly smaller than the CAD, which might also be undesirable. However, the difference was almost negligible, even smaller than 0.01 mm. The relationship between dimensional accuracy, hatch distance, and scan speed is in accordance with what was found by Han et al. [44]. Indeed, they also suggested using higher speed and hatch distance to improve the final dimensional accuracy, especially when fabricating inclined features.

**Figure 12:**
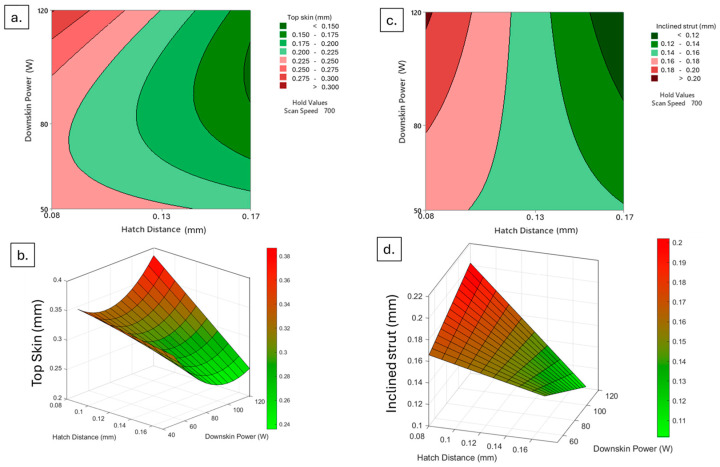
Top skin response to downskin power and hatch distance at constant scan speed of 700 mm/s in 3D (**a**) and contour (**b**) plot; inclined strut response to downskin power and hatch distance at constant scan speed of 700 mm/s in 3D (**c**) and contour (**d**) plot.

Interestingly, the power used for the downskin layers is also reported to influence the final dimensional accuracy, whether on its own or in relation to the hatch distance. The worst results are evidenced when both a lower hatch distance and a higher power are used, as previously observed, because of the excessive energy. However, it can be seen that the lowest energy, meaning lower power and higher hatch distance, does not lead to the best dimensional result (Figure 12), as previously observed.

This is likely because the power employed is insufficient to ensure a proper and complete melting of the powder layer; instead, the melt pool only partially melts the particles, leaving the layers irregular and with unmelted regions. These issues are further exacerbated by the subsequent infill layers, which are exposed to higher energy. On the other hand, an average downskin power or a higher one combined with high hatch distance seems to improve the final surface results, having a more efficiently solidified layer that is not only free of major defects related to overheating effects but also ensures that the layers are completely melted and thus do not suffer from infill laser scans.

#### 3.1.3. Process Parameters Optimisation

**Figure 13:**
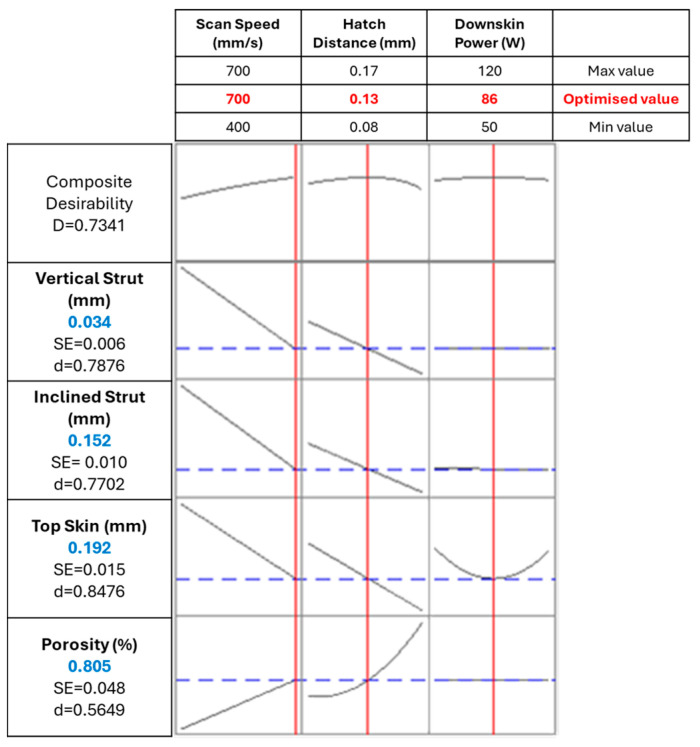
Parameters optimisation: the vertical red lines and red numbers indicate the optimised parameters, while the horizontal blue lines and blue numbers indicate the resulting response.

After having built the model for each response, the optimised parameter set was found, as depicted in the optimisation plot in Figure 13.

The target was set to 0 for all responses to minimise the dimensional deviation and the internal porosity. The model then assigns a value of desirability (d) for each target: the closer to 1, the more favourable the final result is for that response. Having multiple responses that are differently influenced by the parameters, the model measures and maximises a composite desirability D, which takes into account all different desirability. By maximising D, the model defines the parameter set that better compromises the optimisation of all targets. All responses weight and importance were set to 1, aiming for an overall consistent and fair outcome.

The optimised parameter set was then defined, with a scan speed of 700 mm/s, a hatch distance of 0.13 mm, and a downskin power of 86 W. The predicted values can be compared with the ones measured for a sample produced with similar parameter set, which had the same scan speed of 700 mm/s; a slightly higher hatch distance, i.e., 0.13 mm; and a slightly lower power, i.e., 80 W.

As can be observed in Table 3, the results predicted by the model are quite aligned with the experimental values, falling within their standard deviation interval except for the vertical strut. It can also be observed that the standard deviation of the vertical strut is particularly high. Indeed, as previously stated, the vertical strut size showed a standard deviation that was significantly large relative to the mean value due to highly scattered measurements [63,64].

**Figure 14:**
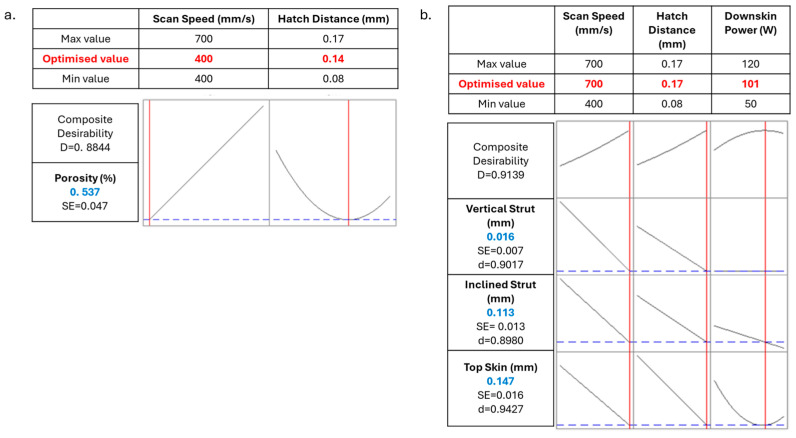
Parameters optimisation of sole porosity (**a**) and dimensional accuracy (**b**); the vertical red lines and red numbers indicate the optimised parameters, while the horizontal blue lines and blue numbers indicate the resulting response.

It can also be observed that the composite desirability is high, though not excellent. Indeed, compromising different responses that showed different relationships to the factors decreases the overall desirability. However, it can be observed that much higher composite desirability results from the optimisation of the sole porosity (Figure 14a) or dimensional accuracy (Figure 14b), with composite desirability around 0.90.

When only the porosity is taken into account, a scan speed of 400 mm/s and a hatch distance of 0.14 mm are predicted to be oriented toward a better final result, i.e., 0.537% of porosity (Figure 14a). No interest is given to the downskin power since it showed no influence on the response in the model. The predicted porosity falls in the range of the standard deviation of the measured result for the closest parameter set, i.e., 0.458 ± 0.098% when using a scan speed of 400 mm/s, a hatch distance of 0.13 mm, and considering a downskin power of 80 W.

Similarly, the parameter set that optimised the dimensional accuracy was identified by the model, with a scan speed of 700 mm/s, hatch distance of 0.17 mm, and downskin power of 101 W. The predicted results reported in Figure 14b are compared with measurements from samples produced at the same scan speed and hatch distance as the prediction but with downskin powers of 80 or 120 W. It can be observed that the predicted values lie within the experimental ranges for both samples, which exhibited an inclined strut deviation of 0.116 ± 0.061 mm and 0.083 ± 0.060 mm and a top skin deviation of 0.142 ± 0.110 mm and 0.175 ± 0.086 mm for 80 W and 120 W, respectively. In general, the sample produced with 80 W downskin power showed mean values in closer agreement with the predicted values, i.e., 0.113 mm for the inclined strut and 0.147 mm for the top skin. However, the vertical strut data show larger discrepancies between predicted and experimental values. Although the predicted value of 0.016 mm falls within the experimental standard deviation range, it differs notably from the measured values of −0.006 ± 0.043 mm and −0.003 ± 0.037 mm for 80 W and 120 W, respectively, which also show significantly high standard deviation. This discrepancy is attributed to the high variability in the measured vertical strut data, which leads to larger uncertainty in these measurements, as already observed for vertical struts.

Since the dimensional accuracy was reported to have a strong correlation with the surface roughness [44], surface roughness was evaluated on selected samples, measuring random inclined struts and downskin of top skin areas. Table 4 reports the roughness parameters of the three samples produced with the parameter sets close to the three optimised ones discussed before.

Upon comparing the three sets, it is clear that surface roughness in these areas improves significantly when the scan speed is increased from 400 to 700 mm/s. This improvement is even more pronounced when the parameters are optimised for dimensional accuracy, by increasing the hatch distance from 0.13 to 0.17 mm, highlighting the strong correlation between surface roughness and dimensional accuracy. The improvement in surface roughness with increased scan speed is also evident in the table found in the Appendix A (Table A2). This table presents the average roughness values Sa, Sz, Sq, Sp, and Sv for samples tested at the same scan speeds. It shows that the surface roughness is worse when slower speeds are used, but it significantly improves when speeds of 600 or 700 mm/s are employed, similarly to the improved dimensional accuracy.

**Figure 15:**
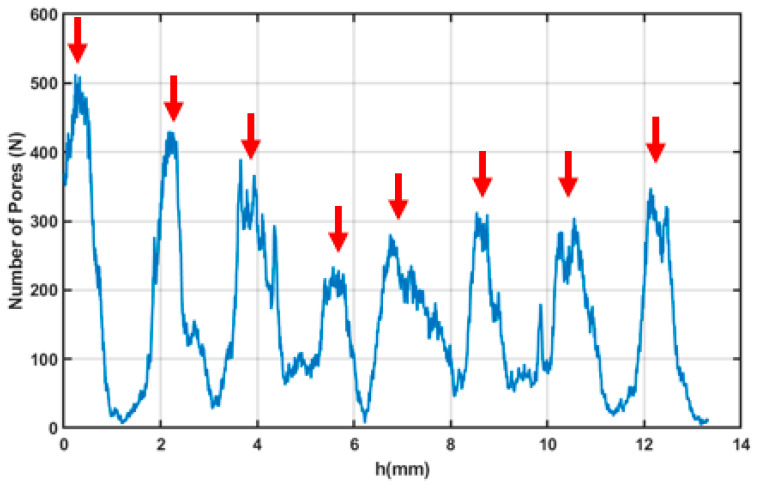
Plot of the number of pores on the sample surface as a function of the section depth, with each point corresponding to a specific height (h) along the sample. The arrows indicate the positions along h where the sample nodes are located.

On the basis of these results, for the following mechanical tests, samples were produced using the parameters set closer to the optimised one, thus using a scan speed of 700 mm/s, a hatch distance of 0.13 mm, and a downskin power of 80 W. X-ray Computed tomography was performed on the sample to analyse its internal porosity distribution. A three-dimensional, section-by-section analysis revealed that pores predominantly accumulate at the nodes of the structure (Figure 15).

**Figure 16:**
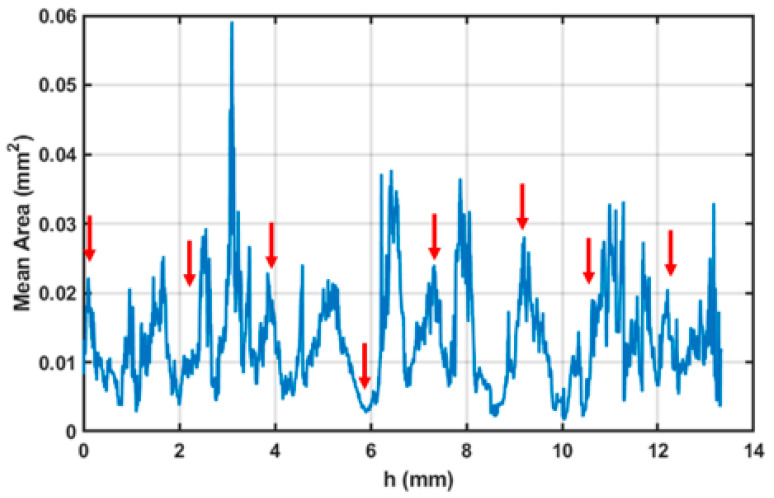
Plot of the mean area of the pores on the sample surface as a function of the section depth, with each point corresponding to a specific height (h) along the sample. The arrows indicate the positions along h where the sample nodes are located.

A notably high concentration was observed at the first node, likely because of its close proximity to the sample base, which may have caused artifacts or pore misidentification. The average pore area was found to range between 0.03 and 0.05 mm^2^ (Figure 16).

**Figure A1:**
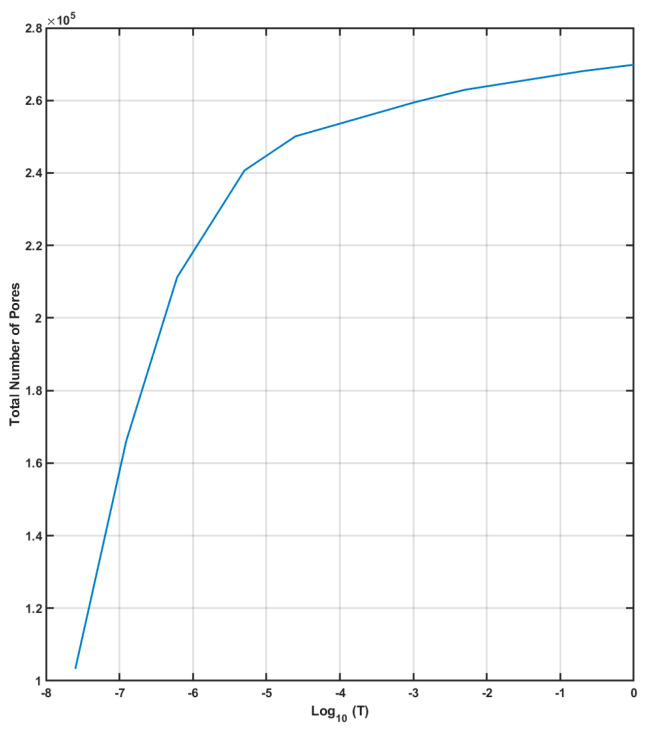
Plot of the total number of pole of the sample in function of the threshold used for the sample mean area in logarithm scale.

The sample area exhibits a complex thermal history, with heat conducted from the surrounding struts influencing the area and potentially causing localised overheating. Such thermal effects have previously been associated with increased powder adhesion in similarly affected regions. To improve the reliability of pore detection, a size-based threshold (T) was implemented in the image processing algorithm to selectively filter out pores exceeding a defined maximum area. Analysis of this filtering approach demonstrated an exponential increase in detected pore count with increasing threshold values (Figure A1, in Appendix A). However, beyond a critical range (approximately 10^−3^–10^−2^ mm^2^), this relationship transitioned to a linear trend. This characteristic response indicates that real pores are predominantly clustered in the 10^−2^ mm^2^ size range, while larger detected features likely represent imaging artifacts or classification errors that are effectively eliminated by the thresholding process. This result confirms the good level of density reached for the optimised samples, with major porosities located in areas subjected to complex thermal and physical phenomena like nodes.

### 3.2. Mechanical Testing

After having picked the desirable set of parameters, the mechanical behaviour of the lattices was analysed. Before testing, it is possible to predict the dominating behaviour based on the cell design by measuring the Maxwell number, which creates a relationship between the type of cell used and the main mechanical behaviour of the structure.

**Figure 17:**
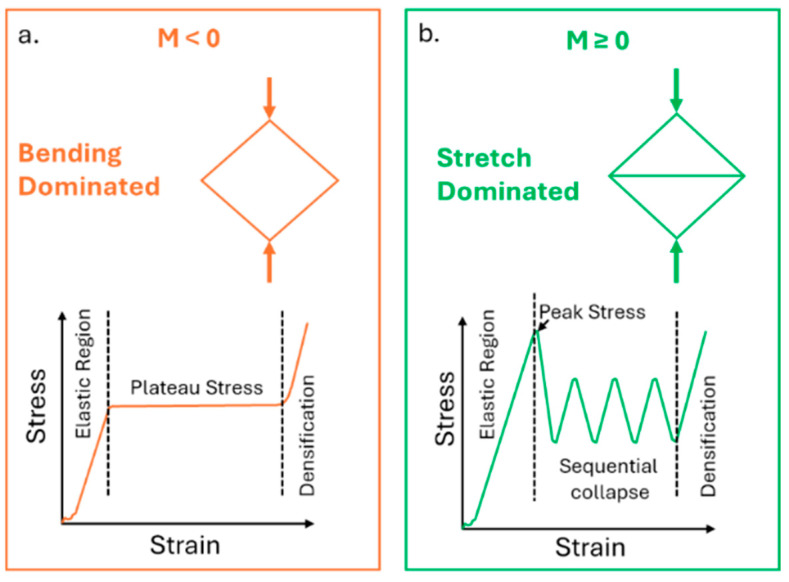
Typical compressive behaviour of a bending-dominated (**a**) and stretch-dominated (**b**) lattice structure and its representative frame.

In fact, as previously stated, lattices can show a bending-dominated behaviour, characterised by lower strength but mostly uniform densification, or a stretch-dominated one, with higher stiffness and yield strength but more prone to sudden failures. Cells exhibiting a bending-dominated behaviour are usually characterised by a negative Maxwell number (Figure 17a); otherwise, they are more prone to display a stretch-dominated behaviour (Figure 17b).

The Maxwell number is expressed as follows:

(3) M=b−3j+6

where *b* is the number of interacting struts, and *j* is the number of nodes. In the samples used in the test, two cells can be recognised: indeed, though the cell is a BCC type, the outer cells also have vertical struts that increase the number of interacting struts. In both cases, 9 nodes are present, while the number of interacting struts is 10/11 for the outer cells and 8 for the inner ones. The final Maxwell number is negative for both types of cells: −10/−11 and −13 for the outer and the inner cells, respectively; therefore, the structure will most probably show a bending-dominated behaviour.

Furthermore, as previously introduced, the Ashby–Gibson model can be used to describe the lattice compressive properties, yield strength $(\sigma_{y}$) and quasi-elastic gradient $\tilde{E}$, as a scaling law, expressed as

(4) $$\sigma_{y}=C_{1}\;\sigma_{y_{s}}{\left(\frac{\rho }{\rho_{s}}\right)}^{n}$$

(5) $$\tilde{E}=C_{2}{\;E}_{s}{\left(\frac{\rho }{\rho_{s}}\right)}^{m}$$

where $\sigma_{y_{s}}$ and $E_{s}$ are the material yield strength and elastic modulus, and $\frac{\rho }{\rho_{s}}$ is the lattice relative density, while C_1_ and C_2_ are the coefficients and n and m the exponential factors that express the relationship. For an open-cell foam exhibiting a bending-dominated behaviour, as could be expected by the cell used in this work, Ashby et al. [14] predicted an exponential factor n of 3/2 and m of 2, a coefficient C_1_ in the range 0.1–1, and C_2_ in the range 0.1–4.

As can be observed by the equation, the Ashby–Gibson model expresses a clear relationship between the lattice compressive behaviour and both the relative density and the material intrinsic properties. Therefore, the analysis of how the lattice compressive behaviour actually relates to the material microstructure and bulk mechanical properties for additively produced lattices is of great interest. Scalmalloy^®^ was considered a good candidate to highlight this relationship since this high-strength alloy is notoriously characterised by radically different behaviours before and after the conventional heat treatment. Indeed, the recommended heat treatment for Al-Sc alloys at 325 °C for 4 h is designed to promote the controlled precipitation of Al_3_Sc without causing any grain growth. Testing Scalmalloy^®^ lattice in the AB and HT conditions using samples with the same structure design and different thermal history allows to verify if the relationships between microstructural changes and mechanical performances influence in the same way the performance of bulk and lattices, as suggested by the Ashby–Gibson scaling law. Tensile properties of bulk samples were used in the present work to represent the material intrinsic properties, similarly to previous research [16,65,66]. Furthermore, the relationship between mechanical properties and relative densities can be explored by testing lattice samples AB and HT with the same conditions but having different strut sizes, thus further broadening the understanding of the reliability of the model.

In the following paragraphs, the results of mechanical tests are reported. At first, the tensile properties of bulk samples were measured in order to define $\sigma_{y_{s}}$ and $E_{s}$. Then, compressive tests were performed on the reference structure with a strut diameter of 1.2 mm to explore the different behaviours before and after the heat treatment. Finally, compressive tests on structures with different strut diameters were performed, observing the relationship of the main properties with the relative density.

#### 3.2.1. Tensile Tests

**Figure 18:**
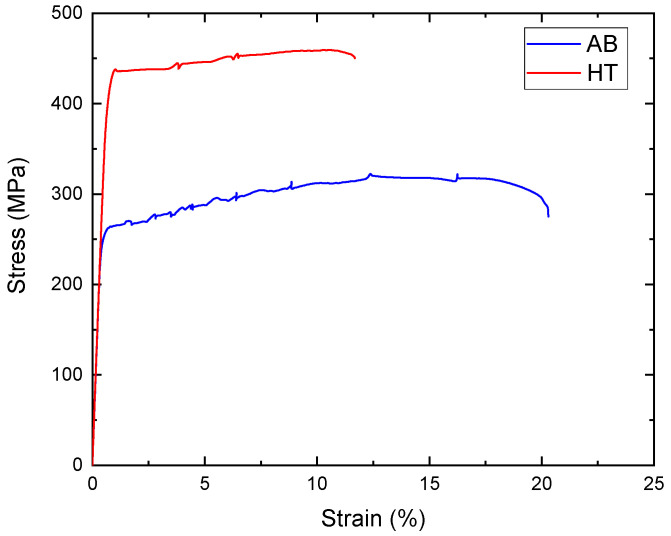
Tensile stress–strain curve of bulk Scalmalloy^®^ in both AB and HT conditions.

Tensile tests were performed to define the yield strength of the alloy $\sigma_{y_{s}}$ and the elastic modulus Es in the AB and HT conditions, and the resulting tensile curves are displayed in Figure 18.

As expected, the heat treatment greatly improved the strength of the alloy. Interestingly, a small amount of scattering in the tensile curves was reported. This scatter is a consequence of the Portevin–Le Chatelier effect, caused by the interaction between dislocations and atoms in solid solution (Mg) or fine coherent precipitates (Al_3_Sc) [32].

The measured $\sigma_{y_{s}}$ increased from 256.38 ± 1.51 MPa for the AB condition to 424.14 ± 2.36 MPa for the HT condition. In contrast, $E_{s}$ was basically unaltered by the heat treatment, having measured values of 68.15 ± 3.34 GPa for the AB condition and 68.87 ± 2.25 GPa for the HT one. Considering the generic Ashby–Gibson model for bending-dominated lattices, it is possible to obtain a range of the predicted mechanical properties of the analysed lattice for the three relative densities, as reported in Table 5.

#### 3.2.2. Compression Tests

**Figure 19:**
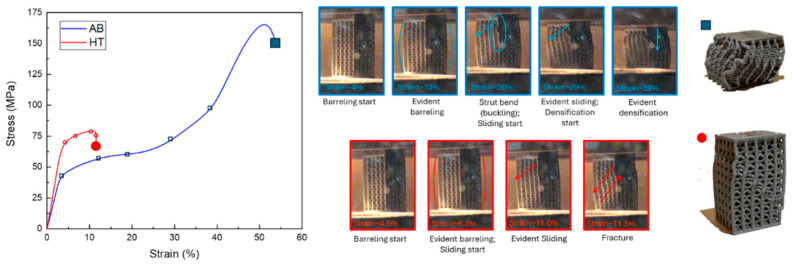
Compressive curves of the lattice structures with strut diameter 1.2 mm, with selected photograms from the test and the final state of the tested sample.

Figure 19 shows the compressive behaviour of the reference lattice with 1.2 mm of strut diameter, i.e., relative density 43%, in both the AB and HT conditions.

It is immediately evident that the lattice compressive properties well-mirror the bulk material tensile properties. Indeed, the AB lattice sample shows lower yield stress and higher ductility, whereas the HT one enhances stress at the expense of deformation before fracture. Comparing the recorded properties with the ones predicted by the model (Table 5), it can be observed that all measured values fell in the predicted range. In particular, the values of $\tilde{E}$ are 1.42 and 2.00 GPa for AB and HT conditions, respectively, whereas values of $\sigma_{y}\;$are, respectively, 39.87 and 62.19 MPa. While the values of $\tilde{E}$ tend to fall closer to the lower end of the range, the values of $\sigma_{y}$ tend to be very close to the average of the prediction, thus with a decent fitting. Interestingly, it is possible to observe a similar enhancement of yield stress after heat treatment for the bulk tensile and lattice compression results. Indeed, the yield strength increases by 65% after heat treatment in the bulk tensile samples, while it increases by 55% in the lattice compressive ones. This similar response to the heat treatment of the two tests, having similar strength enhancement and loss of ductility, further supports the notion that the compressive behaviour is strictly related to the bulk material properties.

Looking in greater detail, different steps of the compressive behaviour of the AB sample can be recognised. After the first elastic region, where no evident deformation could be observed on the lattice, barrelling started occurring at about 4% of deformation, about when the $\sigma_{y}\;$was reached. The barrelling kept occurring until the struts could not stand the stress anymore, and the external vertical struts started bending, failing in buckling mode, as observable at around 20% of deformation. At the same moment as the buckling started, a clear sliding toward the side was recorded since the deformation follows the shear stresses, which are maximum at an angle of around 45°. After about a further 9% of deformation, during which the struts kept bending and the structure slipping, the structure tended to bend on itself, stopping the sliding behaviour and starting the full densification. At the end of the test, a very high level of densification was reached, with the structures completely compacted without having exhibited any failure, showing the expected bending-dominated behaviour.

A different behaviour was observed for the HT condition. After the first elastic region, barrelling was observed, although to a lesser extent than in the AB condition. After about 11.0% of the deformation, the structure began to slip, and after only 0.5% of further deformation, a failure was recorded, with the slip bands at 45° to the loading direction, aligned with the maximum shear stress. It is important to note that the test was interrupted at the first occurring failure; however, the fracture did not result in a complete detachment of the two parts of the structures, as is typically observed in cases of very fragile and abrupt failures [67]. Considering the mechanism of the first fractures observed, it thus can be theorised that a longer test would have led to the typical stretch-dominated behaviour with consequential failures, as observed in other works [68,69], and would have exhibited a higher energy absorption compared to the AB, though characterised by multiple sudden failures instead of more uniform densification.

The behaviour of the HT condition is more compatible with a stretch-dominated behaviour, which contradicts the behaviour expected from the Maxwell number, whereas the AB showed the expected bending-dominated behaviour. This contradiction can be explained by the different thermal histories of the two conditions; indeed, the Maxwell number only takes into account the cell design, but the HT sample underwent a strengthening heat treatment. It can be presumed that the behaviour of the HT alloy is not dominated by the cell design as the AB but by the material microstructure resulting from the heat treatment. In particular, the precipitation of Al_3_Sc caused the strength increase and the loss of ductility, leading to the transition from the bending-dominated behaviour and for the AB condition to the stretch-dominated one. A similar effect of the heat treatment on the dominating compressive behaviour of the lattices was also observed by Banait et al. [68] for an Inconel 718 lattice, who reconducted the behaviour transition to the massive precipitation during heat treatment and the resulting strength and elastic modulus increase.

#### 3.2.3. Ashby–Gibson Model

**Figure A2:**
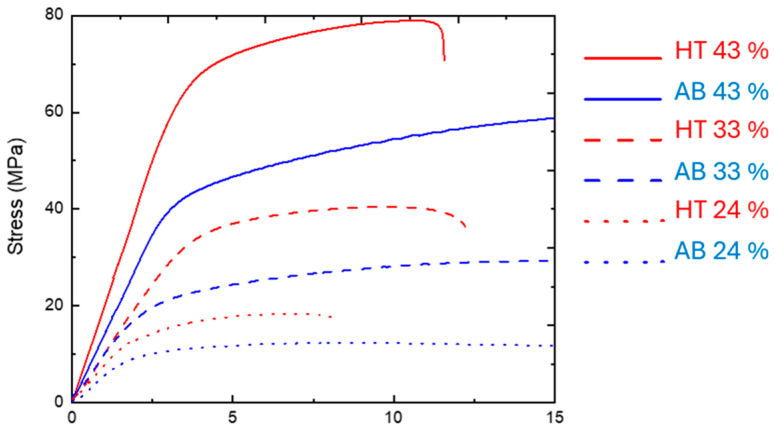
Compressive stress–strain curves of lattice at different treatment conditions and relative densities.

After having tested the structure with 1.2 mm diameter strut, i.e., relative density 43%, two structures with lower densities of 24 and 33% were tested, with the resulting curves reported in Figure A2 and the measured $\tilde{E}$ and $\sigma_{y}$ reported in Table 6. These experimental data were also used to analyse the relationship between mechanical properties and relative density while also performing the comparison and fitting with the Ashby–Gibson model.

Firstly, it can be noted that the properties measured for the AB condition are consistently lower than those for the HT condition, confirming the strengthening effect of heat treatment. Comparing the experimental data with the predicted values from the Ashby–Gibson model in Table 5, it can be observed that all results fall within the predicted range.

**Figure 20:**
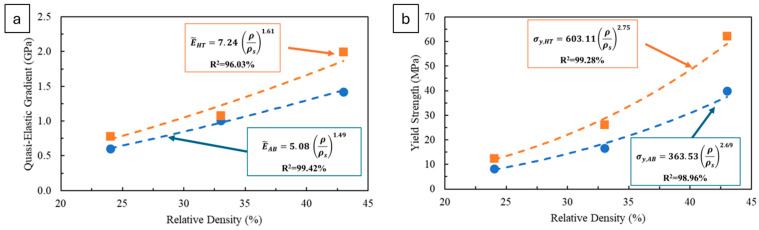
Plotted quasi-elastic gradient (**a**) and yield strength (**b**) versus relative densities in comparison with the predicted behaviour from the Ashby–Gibson model.

However, observing the resulting scaling law for each condition, it is clear that they do not respect the equation suggested by the Ashby–Gibson model. The exponential factor for the $\tilde{E}$ scaling law is 1.49 and 1.61, respectively, for the AB and HT conditions (Figure 20a).

The Ashby–Gibson model predicts an exponential factor of 2, thus slightly higher than the measured ones. On the other hand, the exponential factor measured for the $\sigma_{y}\;$is higher than the model’s 1.5, being 2.69 and 2.75, respectively for the AB and HT conditions (Figure 20b). Nonetheless, the coefficient of determination R^2^ is high in all cases, always higher than 96%, implying that the found scaling laws fit very well with the experimental data even if they are different from the model one.

However, though many authors have reported their experimental data on metallic lattices to fit in the predicted range [18,70,71], it is not uncommon for lattices that have a regular cell repetition and that are produced with metal alloys to exhibit different behaviours compared to the Ashby–Gibson model [17,72]. For instance, the scaling laws observed by Yan et al. [73] for PBFed 316L lattice structures were mostly in accordance with the Ashby–Gibson model but with differences attributable to residual stresses or minor inaccuracies that were more significant for the σ_y_. On the other hand, Li et al. [74] found an exponential factor of 2.7 to describe the fatigue strength of Ti6Al4V lattice structures, which was much higher than the predicted 1.5. Some also found one property exhibiting a behaviour in accordance with the model, while the other did not respect the predicted behaviour [65,67]. The deviations might be explained by the fact that Ashby–Gibson scaling relationships have been derived from extensive experimental data on polymeric foams rather than by the many simplifications used, resulting in a model that is predominantly theoretical and that greatly differs from the reality of the materials, especially for lattice produced by AM, which are characterised by a very peculiar microstructure. Moreover, the model does not account for many other factors, such as process-induced defects or geometry-related stress concentrations. For instance, the porosity concentration at nodes, as shown by the tomography results, can cause localised stress concentrations that are not considered in theoretical models, leading to reduced properties. Generally, porosities such as lack-of-fusion are distributed in homogeneously within the material, resulting in unpredictable failures and variable performance. Other intrinsic defects, including strut waviness, surface roughness, and dimensional inaccuracies caused by the process, may lead to geometrically and dimensionally inaccurate struts, also resulting in relative densities that differ from theoretical values. It has also been reported that compressive properties can be reduced due to texture bias, and this effect is exacerbated by defects located in downskin areas, such as dross on diagonal and horizontal struts, further decreasing structural performance [75]. Additionally, residual stresses induced by the process are not considered in the model, although they may affect the mechanical properties and contribute to deviations from theoretical predictions [73]. Furthermore, the geometry of the structure is not considered in the model apart from it being more stretch or bending dominated, and potential stress concentrations arising from cell geometry are therefore not accounted for.

In conclusion, the Ashby–Gibson model is a powerful model to be used as a first-hand approximation to broadly foresee the lattice behaviour when only knowing the cell design and the employed material. However, the presented results highlight the need to use adjusted models or empirical scaling laws for more precise predictions when dealing with metal lattice structures, particularly considering design features and material conditions. Building on these preliminary findings, further studies should focus on increasing the number of experimental data points to improve model accuracy and enable a more rigorous validation. Additionally, exploring alternative models or analytical approaches could further enhance the reliability and depth of the analysis.

## 4. Conclusions

This study demonstrated the critical role of process parameters in determining the density and dimensional accuracy of lattice structures produced by PBF-LB using the high-performance Scalmalloy^®^ as material, employing statistical tools to identify optimised parameters. The factorial design of experiments and statistical analysis demonstrated that scan speed, hatch distance, and downskin power significantly affect porosity levels and dimensional accuracy as well as surface roughness, with the optimal parameter set identified as 700 mm/s scan speed, 0.13 mm hatch distance, and 80 W downskin power.

Mechanical tests were performed to discuss the influence of heat treatment and relative density on compressive behaviour. Heat treatment substantially enhanced the mechanical properties of the lattice structures, increasing yield strength and causing the failure mode to shift from bending- to stretch-dominated behaviour. This transition underscores the influence of material microstructure in governing deformation mechanisms, potentially outweighing geometric predictions based on the Maxwell number alone. The comparison with the Ashby–Gibson model revealed that the experimental results fell within the predicted ranges; in addition, evident power relationships were found between relative density and compressive properties. However, the scaling laws deviated from the model, particularly for the yield strength evaluation. These discrepancies can be attributed to factors not accounted for by the model, such as local geometric imperfections and process-induced defects.

Overall, this work contributes valuable guidelines for parameter optimisation and a deeper understanding of design–property relationships in additively manufactured Scalmalloy^®^ lattices.

## Figures and Tables

**Table 1:** Scalmalloy^®^ powder chemical composition, as provided by LPW.

	Mg	Sc	Mn	Zr	Si	Fe	Other Elements	Al
%wt	4.77	0.78	0.51	0.27	0.06	0.12	<0.8	Bal.

**Table 2:** Description of factors and respective levels, and responses analysed in the DOE.

Factors				
Name	Level 1	Level 2	Level 3	Level 4
Scan Speed, y (mm/s)	400	500	600	700
Hatch Distance, hd (mm)	0.08	0.13	0.17	/
Downskin Power, Pd, (W)	50	80	120	/
**Responses**				
Porosity			(%)	
(deviation from CAD of) Top skin			(mm)	
(deviation from CAD of) Inclined Strut			(mm)	
deviation from CAD of) Vertical Strut			(mm)	

**Table 3:** Parameters and response values predicted by the model optimisation compared to the those measured from the sample with the closest parameter set.

	Model Optimisation	Experimental Value
Scan speed (mm/s)	700	700
Hatch Distance (mm)	0.13	0.13
Downskin Power (W)	86	80
Porosity (%)	0.805 ± 0.048	0.770 ± 0.058
Top skin (mm)	0.192 ± 0.015	0.213 ± 0.097
Inclined strut (mm)	0.152 ± 0.010	0.165 ± 0.062
Vertical strut (mm)	0.034 ± 0.006	0.048 ± 0.049

**Table 4:** Surface roughness measurements of selected samples.

		Optimised Porosity		Overall Optimisation		Optimised Dimensional Accuracy	
v		400 mm/s		700 mm/s		700 mm/s	
hd		0.13 mm		0.13 mm		0.17 mm	
Pd		80 W		80 W		80 W	
Top skin (µm)	Sq	25.6	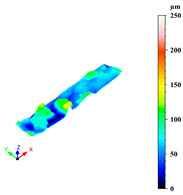	14.8	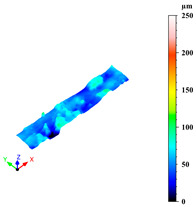	9.5	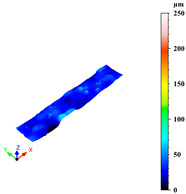
	Sz	152.0		99.1		69.6	
	Sa	19.8		11.5		7.1	
	Sv	66.5		53.3		28.7	
	Sp	85.4		45.8		41.2	
Diagonal Strut (µm)	Sq	14.6	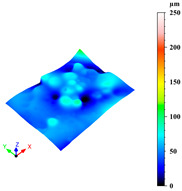	10.9	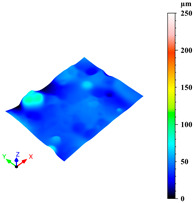	10.7	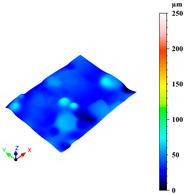
	Sz	121.0		88.7		75.0	
	Sa	11.0		7.5		8.1	
	Sv	48.4		41.3		36.8	
	Sp	72.6		47.4		38.4	

**Table 5:** Yield strength (σ_y_) and quasi-elastic gradient ($\tilde{E}$) as predicted by the Ashby–Gibson model at different treatment conditions and relative densities.

		$\tilde{E}=\left(0.1-4\right)\;E_{s}{\left(\frac{\rho }{\rho_{s}}\right)}^{2}$ [GPa]		* σy=0.1−1 σysρρs32 MPa *	
		AB	HT	AB	HT
**Relative density**	24%	0.40–15.83	0.40–16.04	3.04–30.38	5.02–50.25
	33%	0.74–29.65	0.75–30.03	4.86–48.63	8.04–80.43
	43%	1.26–50.40	1.28–51.04	7.24–72.41	11.97–119.74

**Table 6:** Measured σ_y_ and $\tilde{E}$ at different treatment conditions and relative densities.

		*Quasi-Elastic Gradient* $\tilde{E}$ [GPa]		*Yield Stress* $\sigma_{y}$ [MPa]	
		AB	HT	AB	HT
**Relative** **density**	24%	0.60 ± 0.01	0.78 ± 0.01	8.32 ± 0.11	12.54 ± 0.03
	33%	1.01 ± 0.01	1.08 ± 0.02	16.54 ± 0.09	26.08 ± 1.51
	43%	1.42 ± 0.02	2.00 ± 0.02	39.87 ± 0.04	62.19 ± 0.60

## Data Availability

The original contributions presented in this study are included in the article. Further inquiries can be directed to the corresponding authors.

## Appendix A

**Table A1:** Full results used in the DoE analysis.

	Factors			Responses							
	v (mm/s)	hd (mm)	Pd (W)	Porosity (%)		Deviation of Top Skin (mm)		Deviation of Inclined Strut (mm)		Deviation of Vertical Strut (mm)	
				Av.	Std Dev	Av.	Std Dev	Av.	Std Dev	Av.	Std Dev
A1	400	0.08	50	0.588	0.032	0.303	0.227	0.298	0.259	0.111	0.098
A2	400	0.08	80	1.012	0.046	0.367	0.187	0.246	0.089	0.124	0.089
A3	400	0.08	120	0.579	0.017	0.362	0.152	0.230	0.129	0.073	0.079
A4	400	0.13	50	0.480	0.013	0.234	0.188	0.205	0.109	0.074	0.070
A5	400	0.13	80	0.458	0.098	0.248	0.158	0.264	0.079	0.091	0.062
A6	400	0.13	120	0.440	0.042	0.308	0.127	0.246	0.077	0.076	0.070
A7	400	0.17	50	0.520	0.088	0.354	0.216	0.215	0.105	0.054	0.083
A8	400	0.17	80	0.612	0.030	0.231	0.183	0.246	0.104	0.093	0.066
A9	400	0.17	120	0.616	0.011	0.256	0.191	0.237	0.104	0.076	0.059
B1	500	0.08	50	0.701	0.038	0.366	0.209	0.253	0.070	0.092	0.062
B2	500	0.08	80	0.532	0.038	0.330	0.150	0.291	0.092	0.110	0.052
B3	500	0.08	120	0.953	0.018	0.469	0.140	0.382	0.072	0.158	0.062
B4	500	0.13	50	0.854	0.018	0.318	0.191	0.277	0.091	0.098	0.062
B5	500	0.13	80	0.873	0.074	0.285	0.119	0.263	0.088	0.097	0.043
B6	500	0.13	120	0.679	0.089	0.281	0.093	0.214	0.073	0.048	0.054
B7	500	0.17	50	0.779	0.087	0.342	0.130	0.251	0.090	0.072	0.051
B8	500	0.17	80	0.787	0.026	0.218	0.090	0.216	0.076	0.053	0.052
B9	500	0.17	120	0.702	0.039	0.207	0.076	0.171	0.057	0.041	0.045
C1	600	0.08	50	0.901	0.096	0.280	0.205	0.187	0.088	0.050	0.060
C2	600	0.08	80	0.620	0.014	0.232	0.122	0.236	0.079	0.061	0.065
C3	600	0.08	120	0.651	0.089	0.306	0.178	0.217	0.066	0.062	0.073
C4	600	0.13	50	0.639	0.052	0.247	0.128	0.179	0.064	0.064	0.064
C5	600	0.13	80	0.686	0.100	0.198	0.151	0.180	0.055	0.048	0.059
C6	600	0.13	120	0.696	0.064	0.205	0.098	0.150	0.061	0.033	0.043
C7	600	0.17	50	1.117	0.050	0.236	0.102	0.127	0.054	0.034	0.055
C8	600	0.17	80	1.008	0.053	0.197	0.106	0.180	0.073	0.046	0.027
C9	600	0.17	120	0.924	0.079	0.205	0.106	0.120	0.076	0.049	0.040
D1	700	0.08	50	0.709	0.055	0.257	0.180	0.145	0.064	0.031	0.041
D2	700	0.08	80	0.638	0.024	0.226	0.163	0.139	0.098	0.055	0.049
D3	700	0.08	120	0.834	0.072	0.236	0.097	0.214	0.079	0.061	0.051
D4	700	0.13	50	0.840	0.013	0.270	0.106	0.175	0.078	0.055	0.051
D5	700	0.13	80	0.770	0.058	0.213	0.097	0.165	0.062	0.048	0.049
D6	700	0.13	120	0.735	0.019	0.227	0.100	0.164	0.078	0.028	0.038
D7	700	0.17	50	0.982	0.011	0.205	0.094	0.149	0.055	0.022	0.039
D8	700	0.17	80	0.979	0.016	0.142	0.110	0.116	0.061	−0.006	0.043
D9	700	0.17	120	1.279	0.039	0.175	0.086	0.083	0.060	−0.003	0.037

**Table A2:** Average roughness at different scan speeds.

	Top Skin					Diagonal Strut				
Scan Speed (mm/s)	Sq (µm)	Sz (µm)	Sa (µm)	Sv (µm)	Sp (µm)	Sq (µm)	Sz (µm)	Sa (µm)	Sv (µm)	Sp (µm)
**400**	27.2	144.3	21.7	72.6	89.9	23.3	132.3	17.9	59.1	73.3
**500**	27.6	153.2	22.0	55.5	92.3	22.4	135.0	17.1	65.4	69.6
**600**	19.5	111.0	15.3	44.6	70.2	18.1	106.6	14.7	44.4	62.3
**700**	20.8	113.9	16.0	52.7	67.6	19.1	101.2	15.2	52.3	48.9
